# Consensus Report by the Italian Academy of Osseointegration on the Importance of Peri-Implant Soft Tissues

**DOI:** 10.3390/medicina60091393

**Published:** 2024-08-26

**Authors:** Eriberto Bressan, Giovanni Zucchelli, Grazia Tommasato, Paolo Pesce, Luigi Canullo, Consensus Meeting Group IAO, Maria Gabriella Grusovin

**Affiliations:** 1 Department of Neurosciences, School of Dentistry, University of Padova, Via Giustiniani 2, 35100 Padova, Italy; eriberto@studiobressan.com; 2 Periodontology Unit, Department of Biomedical and Neuromotor Sciences, Bologna University, 40126 Bologna, Italy; giovanni.zucchelli@unibo.it; 3 Department of Biomedical, Surgical, and Dental Sciences, University of Milano, 20122 Milan, Italy; 4 Division of Prosthodontics and Implant Prosthodontics, Department of Surgical Sciences, University of Genova, Largo R. Benzi 10, 16132 Genova, Italy; paolo.pesce@unige.it (P.P.); luigi.canullo@gmail.com (L.C.); 5 Department of Dentistry, Università Vita-Salute San Raffaele, 20132 Milan, Italy; gabri.grusovin@tiscali.it

**Keywords:** collagen, dental implant, keratinized tissue, zirconia, titanium, matrices

## Abstract

*Background and Objectives*: The influence of the quantity and quality of peri-implant soft tissue on implant health and long-term maintenance is controversial. This consensus aimed to assess the importance of peri-implant soft tissue by analyzing four aspects: the role of keratinized mucosa (KM), the efficacy of specific collagen matrix, the influence of abutment material, and soft-tissue thickness. *Materials and Methods*: Active members of the Italian Academy of Osseointegration (IAO) participated in the consensus. Four systematic reviews were conducted, and their results were discussed to provide guidelines on the importance of soft tissue around implants. The first review evaluated the effect of KM on soft-tissue health, peri-implant bone loss, and patient-related variables. The second one analyzed if there was a specific type of matrix that provided better results in terms of peri-implant buccal soft-tissue thickness and keratinized mucosa width compared to autogenous soft-tissue graft. The third review evaluated the influence of different abutment materials on the soft tissues, and the fourth assessed the effect of soft-tissue thickness on peri-implant marginal bone loss (MBL). *Results and Conclusions*: The agreements reached by the assembly were as follows: the presence of supra-periosteal keratinized tissue is considered to favorably influence peri-implant health and aesthetics but had no relation to preventing bone crest resorption unrelated to infection. It facilitates patient cleaning around implants and reduces patient-reported pain. The free gingival graft (FGG) is considered the best in terms of supra-periosteal KM increase. Connective tissue grafts (CTG) perform better than volume-stable collagen matrices to increase soft-tissue thickness. Collagen matrices reduce surgical time and patient morbidity and can give better camouflaging. The influence of abutment material (titanium or zirconia) on MBL remains controversial, and no conclusion could be reached on this issue. Peri-implant soft-tissue health and recession seem not to be influenced by abutment material, but data are limited to zirconia and titanium. Although this systematic review highlighted the absence of a correlation between soft-tissue thickness and MBL, the assembly failed to find a consensus on this issue.

## 1. Introduction

The success of dental implants is undeniably dependent on biological principles related not only to proper osseointegration at the bone–implant interface but also to adequate and stable soft-tissue integration around the implants [1]. Despite the similarities in the histologic and clinical features between the periodontium and peri-implant supporting structures, there are fundamental differences between teeth and implants in their anchorage and attachment to the surrounding tissues. One key difference is that no periodontal ligament, cementum, and bundle bone (alveolar bone proper) are present around dental implants, resulting in direct contact between the peri-implant bone and the implant surface [2].

Regarding histological features, the peri-implant mucosa consists of a well-keratinized oral epithelium at the outer surface, which is continuous with a sulcular epithelium lining the lateral aspect of the gingival sulcus, similar to the epithelial structure and arrangement of the natural dentition. The inner-lining epithelial attachment of the peri-implant mucosa resembles the junctional epithelium of the teeth in its histological characteristics. It is believed that the formation of this barrier epithelium facing the implant is a natural result of wound healing, initiated at 1–2 weeks and established after 6–8 weeks of healing. The apical cells of the barrier epithelium terminate approximately 1–1.5 mm coronal to the bone crest and are separated from the bone by a noninflamed, collagen-rich, cell-poor connective tissue zone [1,2].

While the peri-implant mucosal connective tissue attachment is clinically and histologically similar to that of teeth, the main difference is observed in the cellular composition and fiber orientation. The connective tissue surrounding the implant is in direct contact with the implant surface and contains a dense network of collagen fibers originating from the periosteum of the peri-implant bone crest, extending to the mucosal margin. These fibers are oriented in a direction parallel to the implant/abutment surface, whereas the attachment of connective tissue to teeth involves collagen fibers inserting into the root cementum in a perpendicular direction [1,2].

The peri-implant epithelium and connective tissue together form a mucosal seal that acts as a barrier to separate dental implants from the oral environment. However, this seal is created through the “adaptation” rather than an “attachment” of the mucosa to the implant/abutment surface. Adhesive structures, such as the internal basal lamina and hemi-desmosomes of the peri-implant junctional epithelium, are scarce and significantly weaker than those found in the periodontal attachment and are only present in the lower part of the peri-implant epithelium–implant interface [1].

This adaptation is crucial in preventing the entrance of oral microorganisms, which can lead to biological complications such as peri-implant mucosa inflammation, bleeding, swelling, pain, pocket formation, peri-implant mucosa recession, bone resorption, and ultimately implant failure [1].

The vascular structure of the peri-implant mucosa begins exclusively from the final branches of larger vessels of the supra-periosteum from the outer (buccal) border of the bone ridge, since the vascular plexus of the periodontal ligament, proper of natural teeth, is missing. Blood vessels lateral to the junctional epithelium in peri-implant mucosa present a “crevicular plexus” and are continuous with the supra-periosteal vessels, similarly to the respective gingival vascular structure. However, the vascular supply proper of the richly vascularized connective tissue adjacent to the root cementum around natural teeth is almost entirely missing in the corresponding peri-implant tissue. This “inflammation-free scar tissue” may impair the defense system of tissues surrounding dental implants and make the peri-implant mucosal tissues more vulnerable to bacterial challenge [2].

In conclusion, the orientation of the fibers and the reduced cellularity and vascularity in the peri-implant connective tissue could make it more susceptible to the initiation and progression of inflammatory disease.

In 2017, the World Workshop on the Classification of Periodontal and Peri-Implant Diseases and Conditions introduced the concept of the “periodontal phenotype”. Building upon this, Avila-Ortiz and colleagues described the “peri-implant phenotype” in 2020 as the morphological and dimensional features of the tissues surrounding and supporting osseointegrated implants [3]. This phenotype includes four components, peri-implant keratinized mucosa width (KMW), mucosal thickness (MT), supra-crestal tissue height (STH), and peri-implant bone thickness (PBT), which are all site-specific and may change over time in response to environmental factors.

Keratinized mucosa width (KMW) is the apico-coronal height of keratinized soft tissue from the mucosal margin to the mucogingival junction. Sometimes, it may be also completely absent, and its optimal amount in terms of functional and aesthetic outcomes is yet to be determined, though an “adequate KMW” has been proposed to be ≥2 mm.

Mucosal thickness (MT) is the horizontal dimension of the peri-implant soft tissue, which may or may not be keratinized. Its optimal cut-off value is also not established, though has been proposed to categorize it in “thin MT” if <2 mm and thick MT if ≥2 mm.

Supra-crestal tissue height (STH) is the vertical dimension of the soft tissue surrounding a dental implant from the mucosal margin to the crestal bone. Different from KMW and MT, STH can also be evaluated proximally. A categorization into “short” STH if <3 mm and “tall” STH if ≥3 mm has been proposed.

Peri-implant bone thickness (PBT) is the horizontal dimension of the osseous tissue supporting an osseointegrated implant, which may vary at different apico-coronal heights relative to the bone crest around an implant. There is limited clinical evidence to establish a minimum threshold of bone thickness, but “thin” PBT is categorized as <2 mm, and “thick” PBT is considered to be ≥2 mm [3].

In order to create or re-create adequate soft-tissue volumes around dental implants, several techniques have been described in recent decades, and autogenous grafts (such as connective tissue grafts—CTG and free gingival grafts—FGG) still represent the best solution. However, increased morbidity and discomfort, longer operating times, surgical skills, and limited quantities of grafts represent the main drawbacks of autogenous grafts. To overcome these disadvantages, various types of matrices have been placed on the market in recent years. Such matrices can be divided into two main groups: (a) human-based tissue derivatives and (b) animal-based tissue derivatives [4].

The long-term stability of implants can also be influenced by the seal between the peri-implant mucosa and the implant abutment. In fact, this close contact can prevent the development of mucositis and peri-implantitis, which can cause marginal bone loss over time [5]. For this reason, different abutment materials were proposed and are being studied. The most commonly used material is titanium, but alumina, gold-hued titanium, and zirconia have become alternatives with good aesthetic properties.

The present Consensus Conference aimed to assess the role of peri-implant soft-tissue constitutive features on peri-implant health, focusing on four aspects. First, it examined the effect of keratinized mucosa on peri-implant soft-tissue health, peri-implant bone loss, and patient-related variables. Second, it analyzed the efficacy of different types of matrices for enhancing peri-implant buccal soft-tissue thickness and keratinized mucosa width in case of soft-tissue deficiency, compared to autogenous soft-tissue grafts. Third, it evaluated the influence of different abutment materials on the soft tissues, and finally, it examined the effect of mucosal thickness on the peri-implant marginal bone level.

## 2. Materials and Methods

Active members of the Italian Academy of Osseointegration (IAO) took part in the Consensus Conference aimed at providing guidelines on the importance of soft-tissue quality around dental implants. Prior to the meeting, four systematic reviews were conducted, and their conclusions were evaluated. The reviews followed the PRISMA guidelines (http://www.prisma-statement.org/) and the review protocols were registered in PROSPERO (submission Nos. CRD42021231674—accessed on 17 January 2021; CRD42021248859—accessed on 14 May 2021; CRD42021234431—accessed on 9 March 2021; CRD42021235324—accessed on 15 March 2021) [4,5,6,7].

The first systematic review analyzed the effect of keratinized mucosa on peri-implant health and patient-reported outcome measures [6]. The focused question was as follows: “In patients having at least one implant-supported restoration under functional loading for at least 6 months does the presence of keratinized mucosa influence soft-tissue health, bone levels, aesthetics and patient-related variables around implants against the null hypothesis of no influence?”

The focused question was established according to the PICO-T strategy:

Patients (P): healthy patients with at least one dental implant.
Intervention (I),
Comparison (C): studies comparing two groups of patients with presence or absence of KM or with KM < 2 mm or ≥2 mm.
Outcome (O): implant failures, pain, patient satisfaction regarding aesthetics, quality of life, bleeding on probing (BOP), and biological complications. Secondary outcomes were as follows: probing pocket depth (PPD), gingival index (GI), plaque index (PI), marginal bone loss (MBL), and soft-tissue recession (REC).
Time (T): at least 6 months of follow-up.

An ad hoc search string was adapted to each database, and the research was conducted on MEDLINE via PubMed, Cochrane Central Register of Controlled Trials (CENTRAL), and EMBASE (“dental implants”[mh] OR “dental implantation”[mh] OR ((“implant”[tiab] “OR “implants”[tiab]) AND (dental[tiab] OR oral[tiab] OR tooth [tiab]))) AND (“mouth mucosa”[mh] OR“((“peri-implant”[tiab] OR “masticatory”[tiab] OR “attached”[tiab] OR “keratinized”[tiab] OR ”KT”[tiab]) AN“ (“mucosa”[tiab] OR “gingiva”[tiab]))).

The last electronic search was carried out in May 2021.

The second systematic review analyzed the soft-tissue augmentation techniques [4], and the focused question was as follows: “In case of soft tissues deficiencies around dental implants, is there a specific type of matrix which provides greater results in terms of peri-implant buccal soft tissue thickness and keratinized mucosa width compared to autogenous soft tissue graft?”

The focused question followed the PICO-T strategy:

Patients (P): patients with lack of keratinized tissue or mucosal defects around dental implants in need of soft-tissue augmentation.
Intervention (I): collagen matrices placed around dental implants to increase soft-tissue thickness or keratinized mucosa width or both.
Comparison (C): autogenous soft-tissue graft placed around dental implants.
Outcome (O): volumetric and dimensional changes of soft-tissue thickness and keratinized mucosa width following peri-implant soft-tissue augmentation.
Time (T): at least 1 month from intervention.

An ad hoc search string was adapted to each database, and a literature search was carried out using electronic databases including MEDLINE (PubMed), EMBASE, Cochrane Central Register of Controlled Trials, Scopus, and Web of Science: ((“soft tissue” AND “augmentation” AND “tooth implant”) OR “peri implant”) AND (“collagen matrix”). The electronic search was carried out in April 2021, and an update was undertaken in April 2023.

The third systematic review analyzed the effect of the abutment materials on tissue health and stability [5], and the focused question was as follows: “What is the effect of the abutment material on soft tissue health and stability?”

The focused question followed the PICO-T strategy:

Patients (P): healthy patients with abutments connected to dental implants.
Intervention (I): any abutment material different from titanium.
Comparison (C): titanium abutments.
Outcome (O): MBL. Secondary outcomes: PI, BOP, PPD, and REC.
Time (T): at least 6 months follow-up after abutment connection.

A literature search was conducted through electronic databases (MEDLINE (PubMed), Cochrane Central Register of Controlled Trials, and Scopus) using an ad hoc search string that was adapted to each database: (“dental implants”[MeSH Terms] OR (“dental”[All Fields] AND “implants”[All Fields]) OR “dental implants”[All Fields] OR (“dental”[All Fields] AND “implant”[All Fields]) OR “dental implant”[All Fields]) AND (“abutment”[All Fields] OR “abutment s”[All Fields] OR “abutments”[All Fields]) AND (“titanium”[MeSH Terms] OR “titanium”[All Fields] OR “titaniums”[All Fields]) AND (“zirconia”[All Fields] OR “zirconias”[All Fields] OR “zirconium oxide”[Supplementary Concept] OR “zirconium oxide”[All Fields] OR “zirconia”[All Fields] OR “gold”[All Fields] OR “PEEK”[All Fields]). The last electronic search was carried out in February 2022.

The fourth systematic review analyzed the effect of soft-tissue thickness on marginal bone level [7] around dental implants and the focused question was as follows: “What is the effect of soft tissue thickness on peri-implant bone loss assessed by RCTs/CCTs in initial bone remodeling?”

The focused question followed the PICO-T strategy:

Patients (P): healthy patients with at least one dental implant.
Intervention (I): implant placement and soft-tissue healing.
Comparison (C): thickness of the surrounding soft tissues.
Outcome (O): marginal bone level changes.
Time (T): min 10 months–max 14 months.

A literature search was conducted through electronic databases (MEDLINE (PubMed), Cochrane Central Register of Controlled Trials, Web of Science and Scopus) using an ad hoc search string that was adapted to each database: ((dental implants) OR (dental implantation) OR (dental prosthesis implant-supported) OR (oral implants) OR (endosseous implants) OR (implant restoration) OR (osseointegrated implants)) AND ((clinical outcomes) OR (early bone loss) OR (marginal bone loss) OR (bone level changes) OR (marginal bone level) OR (marginal bone resorption) OR (marginal bone remodeling) OR (marginal bone preservation) OR (crestal bone level) OR (crestal bone loss) OR (crestal bone resorption)) AND ((tissue thickness) OR (tissue biotype) OR (tissue phenotype)). The last electronic search was carried out in May 2022.

Inclusion and exclusion criteria for each review are shown in Supplementary Table S1.

The flowchart of the study selection process divided into the four reviews are presented in Supplementary Figures S1–S4.

## 3. Results

The main results of the systematic reviews are summarized in Table 1.

The results of the systematic reviews were discussed on 15/16 October 2021 in the IAO consensus meeting. The questions proposed to the assembly are presented in Table 2, together with the results of the voting.

Concerning the influence of keratinized mucosa on soft-tissue health and patient-related outcomes, the assembly agreed that keratinized mucosa around at least one single implant should be more precisely defined as supra-periosteal keratinized mucosa (attached to bone and do not present mobility due to muscular traction) and crestal keratinized mucosa (that can be movable). Even if the results of the review are controversial, the presence of supra-periosteal keratinized tissue is considered to favorably influence peri-implant health, reducing the presence of peri-implant inflammation (mucositis and peri-implantitis) and the aesthetic of prosthetic restorations, while it had no relation to preventing bone crest resorption unrelated to infection. In addition, both supra-periosteal and crestal keratinized mucosa positively influence the ability and ease of cleaning around implants and the presence of pain reported by the patient.

Concerning soft-tissue deficiencies around implants, free gingival graft (FGG) is considered to be the best in terms of keratinized mucosa (KM) increase. Volume-stable collagen matrices can be used to increase soft-tissue thickness, while a connective tissue graft (CTG) seems to give better outcomes than the volume-stable collagen matrices. In terms of PROMS, both the free gingival and connective tissue grafts, with respect to the collagen matrix, increase the risk of worse camouflaging with the surrounding tissue, surgical time, and patient morbidity.

In periodontally healthy patients, the influence of the abutment material (titanium or zirconia) on crestal bone loss remains controversial, and no conclusion could be reached on this issue. Peri-implant soft-tissue health and soft-tissue recession seem not to be influenced by the abutment material (titanium or zirconia).

Concerning the importance of soft-tissue thickness around implants, while the aforementioned review indicated that the initial soft-tissue thickness failed to present any influence on marginal bone loss after a short follow-up period, the assembly could not confirm this assumption on the basis of the active members’ clinical experiences, and it was not possible to draw definitive conclusions. More studies were advocated on this specific topic.

Limits of each review are shown in Supplementary Table S2.

## 4. Discussion

The present consensus focused on the factors and features related to peri-implant soft tissues, potentially affecting their health and stability over time and, when possible, patient-related outcomes. Four aspects, in particular, were considered: keratinized tissue, graft materials (collagen matrix vs. autogenous collagen), abutment materials, and soft-tissue thickness.

The first aspect investigated was the importance of the keratinized tissue in relation to peri-implant health, esthetics, and patient-related variables. Presence of KM ≥ 2 mm was associated with less marginal inflammation (GI, mGI, BI, msBi, and Bop), plaque accumulation (PI and mPI), recession, biological complications, and less soreness or discomfort during oral hygiene procedures, while no differences were found for implant failures, PPD, and bone levels. These results were similar to the ones reported by other systematic reviews on this topic [63,64,65,66], even if differences in inclusion criteria, methods of analysis, and outcomes analyzed were present. The results should be analyzed with caution since only one RCT and only two studies at low risk of bias were included, and data at implant level, high heterogeneity between studies, and few long-term follow-ups were present. In addition, the differences between groups were small, challenging their clinical significance, and they were even smaller if one study that included patients with poor compliance was excluded. One reason behind the small differences present between groups could lie in the choice of the cut-off for sorting the groups and in the absence of distinction between attached or not-attached keratinized mucosa. This issue was considered important by the assembly, which agreed that a more precise definition should be applied in future studies. In particular, keratinized mucosa should be more precisely defined as supra-periosteal keratinized mucosa (attached to bone, which does not present mobility due to muscular traction) and crestal keratinized mucosa (which can be movable). This consideration led to the consensus conclusion that the presence of supra-periosteal keratinized tissue favorably influences peri-implant health and the esthetics of prosthetic restorations, while it had no relation in preventing bone crest resorption unrelated to infection. Patient-related variables were positively influenced by the presence of KM.

The second review used a network meta-analyses to evaluate the efficacy of different collagen matrices compared with autologous soft-tissue grafts when soft-tissue deficiencies were present. No other network meta-analyses were performed on this issue, so comparison with other reviews is difficult. The validity of the results was limited by the number of included studies [23,24,25,26,27,28,29,30,31,32,33,34,35,36,37,38], the presence of a high heterogeneity between studies, and the inclusion of CCTs with a higher risk of bias in the analysis [34,35,36,37,38]. In addition, the studies included presented a high variability in the number of included patients, surgical techniques, timing to evaluate the soft-tissue augmentation, and methods to evaluate tissue thickness. The conclusion reached by the assembly were in line with the results of the review. While FGG was considered to be the first choice when keratinized mucosa height should be increased, even if the esthetics could be better with a collagen matrix (XCM), volume-stable collagen matrices (VCMX) can be used to increase soft-tissue thickness, but CTG gives the best result in this context. Morbidity with an autologous graft is higher, so more studies on biomaterials are advocated using standardized methods of evaluation and a higher number of patients included.

The third aspect analyzed by the consensus was to detect the correlation between peri-implant tissue health and stability and abutment materials. The review presented some limitations due to the heterogeneity of the studies included, the study designs, the analyses of MBL and PI, clinical procedures, and the different follow-ups. Zirconia performed better, followed by titanium in most of the outcomes, but more studies are needed to obtain more reliable conclusions. According to the systematic review presented, the assembly agreed to consider titanium and zirconia abutments with similar clinical outcomes in terms of soft-tissue health. This was also in agreement with other already published systematic reviews with meta-analyses [67,68]. The assembly, in fact, declared that the choice between the two materials mostly depends by the esthetic requests and the gingival phenotype [69].

The fourth aspect of the consensus was to investigate the importance of supra-crestal soft-tissue height correlated to the marginal bone levels. The systematic review organized for the proceedings of the consensus clearly failed to reveal any direct correlation between these two variables. This was in agreement with a previous systematic review [70]. Additionally, the use of trial sequential analysis (TSA) tools allowed us to understand the real weight of each included study, but it showed that the results should be considered with caution, since the threshold of requiring information size (RIS) was more than threefold higher than the number of patients included in the meta-analysis. However, although the evidence from the systematic review highlighted the absence of a correlation between supra-crestal soft-tissue height and marginal bone level, the assembly failed to find a consensus. Following this line, the assembly suggested the performance of new clinical trials focusing on this topic able to exclude the effect of confounding factors such as preparation-related or prosthetic-related bone resorption.

## 5. Conclusions

The presence of supra-periosteal keratinized tissue could favorably influence peri-implant health, the aesthetic of prosthetic restorations, but not the bone crest resorption unrelated to infection. Supra-periosteal keratinized mucosa facilitates patient cleaning around implants and reduces patient reported pain. A free gingival graft is considered to be the best in terms of supra-periosteal keratinized mucosa increase, while a connective tissue graft performs better than volume-stable collagen matrices to increase soft-tissue thickness. A collagen matrix reduces surgical time and patient morbidity and can give better camouflage with surrounding tissues.

Peri-implant soft-tissue health and recession seem not to be influenced by abutment material, but data are limited to zirconia and titanium. The importance of soft-tissue thickness on initial bone remodeling remains controversial.

## Figures and Tables

**Table 1:** Main results for each review.

Review Topic	Included Studies	Main Results
The Effect of Keratinized Mucosa on Peri-Implant Health and Patient-Reported Outcome Measures: A Systematic Review and Meta-Analysis	3214 were screened and 15 of them were included [8,9,10,11,12,13,14,15,16,17,18,19,20,21,22].	No statistically significant differences between the presence of KM ≥2 mm and <2 mm were found for implant failure, PPD, or bone loss. BoP resulted in being significantly lower in the KM ≥2 mm group only in the three prospective studies. Less statistical marginal inflammation, plaque accumulation, and recession were associated with the presence of KM ≥2 mm, but the differences were clinically small. More biological complications were described in the no KM/KM <2 mm group, but the reduced number of cases does not allow us to draw any conclusions. Although a meta-analysis could not be performed, a consistent trend toward the worst pain/discomfort in KM <2 mm was observed. These results should be considered with caution, since most of the studies were at a moderate and high risk of bias, follow-ups were short, and data were given at the implant level. Furthermore, most of the included patients had low plaque levels and PPD values, so these results may not be valid for patients with erratic compliance that presented as having higher benefits from KM presence.
Autogenous graft versus collagen matrices for peri-implant soft tissue augmentation. A systematic review and network meta-analysis	405 articles were screened, and after full-text evaluation, 11 randomized controlled trials (RCTs) and 5 controlled clinical trials (CCTs) were included [23,24,25,26,27,28,29,30,31,32,33,34,35,36,37,38].	Connective tissue graft (CTG) demonstrated better performance in all the comparisons and the free gingival graft (FGG) showed itself to be the best graft to increase KM (medium term). Adermal matrix (ADM) and volume-stable collagen matrix (VCMX) may be used to increase SHT with lower patient morbidity. RCTs studying this topic with larger sample sizes are needed to better elucidate the effects of different matrices on soft-tissue augmentation. RCTs comparing the use of soft-tissue grafts or matrices before or after implant placement are necessary to provide clinical recommendations.
Effects of abutment materials on peri-implant soft tissue health and stability: A network meta-analysis	The research generated 1437 articles, of which 18 were included [39,40,41,42,43,44,45,46,47,48,49,50,51,52,53,54,55,56].	Zirconia abutments seem a viable alternative to the use of classical titanium abutments. However, due to the great heterogeneity of the studies, more clinical studies are needed to obtain more robust conclusions.
Influence of soft tissue thickness on marginal bone level around dental implants: A systematic review with meta-analysis and trial-sequential analysis	The research generated 186 articles, of which 6 were included [57,58,59,60,61,62].	The evidence of peri-implant bone remodeling due to initial soft-tissue thickness is confirmed, as the meta-analysis demonstrates a distinction between thin and thick tissue condition. It should be emphasized that, from a clinical point of view, this result must be taken into careful consideration and that neither post-extraction implant placement nor immediate-loaded implants were included in the review.

**Table 2:** Consensus question (answers chosen by the assembly are highlighted in bold).

Questions	Possible Answers
In patients having at least one implant-supported restoration under functional loading for at least 6 months, does the presence of keratinized (vestibular and lingual) mucosa influence the presence of soft-tissue inflammation (mucositis)?	**Yes** No
In patients having at least one implant-supported restoration under functional loading for at least 6 months, does the presence of keratinized (vestibular and lingual) mucosa influence the presence of bone crest resorption unrelated to infection?	Yes **No**
In patients having at least one implant-supported restoration under functional loading for at least 6 months, does the presence of keratinized (vestibular and lingual) mucosa influence the aesthetics of the prosthetic restoration?	**Yes** No
In patients having at least one implant-supported restoration under functional loading for at least 6 months, does the presence of keratinized (vestibular and lingual) mucosa influence the ability and ease of cleaning around implants and the presence of pain reported by the patient?	**Yes** No
In case of soft-tissue deficiencies around dental implants, a free gingival graft seems to be the best in terms of keratinized mucosa increase.	**Yes** No
In case of soft-tissue deficiencies around dental implants, volume-stable collagen matrices can be used to increase soft tissue.	**Yes** No
In case of soft-tissue deficiencies around dental implants, a connective tissue graft (CTG) seems to achieve a better outcome than the volume-stable collagen matrices.	**Yes** No
In case of soft-tissue deficiencies around dental implants in terms of PROMS, both the free gingival graft and connective tissue graft (CTG), with respect to the collagen matrix, increase the risk of worse camouflaging with the surrounding tissue, increase the surgical time, and increase patient morbidity.	**Yes** No
In healthy patients with abutments connected to dental implants for at least 6 months, does the abutment material influence the soft-tissue recession around implants?	Yes **No**
In healthy patients with abutments connected to dental implants for at least 6 months, does the abutment material influence the crestal bone loss around the implant?	Yes No **It is not possible to draw definitive conclusions**
In healthy patients with abutments connected to dental implants for at least 6 months, does the abutment material influence the soft-tissue health of peri-implant tissues?	Yes **No**
Does the thickness of the soft tissues surrounding an implant influence the peri-implant initial bone remodeling?	Yes No **It is not possible to draw definitive conclusions**

## Supplementary Materials

The following supporting information can be downloaded at: https://www.mdpi.com/article/10.3390/medicina60091393/s1, Table S1. inclusion and exclusion criteria for each review; Table S2. limits of each review; Figure S1. Literature research process—first review (effect of keratinized mucosa); Figure S2. Literature research process—second review (effect of matrices vs autogenous grafts); Figure S3. Literature research process—third review (effect of the abutment materials); Figure S4. Literature research process—fourth review (effect of soft tissue thickness on peri-implant bone loss).
