# Supplementary material for: Consensus Report by the Italian Academy of Osseointegration on the Importance of Peri-Implant Soft Tissues
Source: Medicina (Kaunas). 2024 Aug 26;60(9):1393. doi: 10.3390/medicina60091393 (PMC11433715; doi:10.3390/medicina60091393)
Supplement: Supplementary file 1 [file medicina-60-01393-s001.zip › medicina-3136591-supplementary.pdf]

Supplementary Materials

Table S1. inclusion and exclusion criteria for each review.

**REVIEW # 1**	**Eligibility criteria**	• Evaluation of soft tissue thickness or volume changes after soft tissue augmentation<br>• Assessment of keratinized mucosa width
	**Exclusion criteria**	• Studies evaluating only hard tissue dimension and volume changes were excluded.<br>• Studies performed on animal models were excluded.
**REVIEW # 2**	**Eligibility criteria**	• Studies with data on bone loss and periodontal parameters that included a 6-month follow-up after abutment connection
	**Exclusion criteria**	• Case reports, animal studies and *in vitro* studies
**REVIEW # 3**	**Eligibility criteria**	• Randomized controlled trials, cohort, cross-sectional and case–control human studies<br>• Studies comparing two groups of patients with a presence or absence of KM, or with KM < 2 mm or ≥2 mm<br>• At least 6 months of follow-up<br>• Reports of correlation with at least one of the outcome measures considered
	**Exclusion criteria**	• Retrospective cohort and cross-sectional studies<br>• No full text availability<br>• Inability to use the KM data or analysis provided
**REVIEW # 4**	**Eligibility criteria**	• Randomized clinical trials (RCTs)<br>• Clinical Trials and Controlled clinical trials (CCTs)<br>• Marginal Bone Level clearly described<br>• Human studies
	**Exclusion criteria**	• Studies with post extraction implant placement<br>• Studies with prosthetic immediate loading (<3 months) Studies reporting hard or soft tissue graft

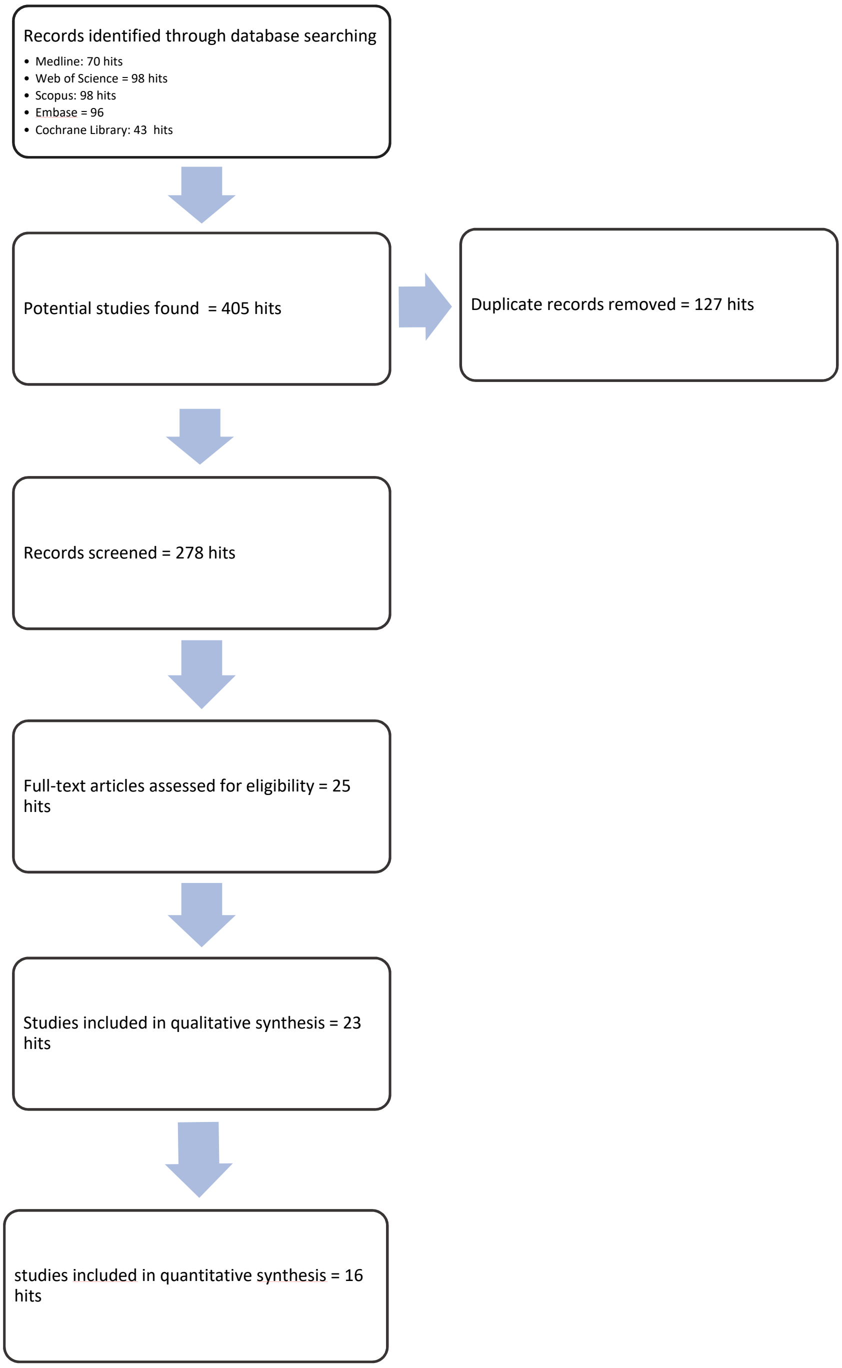

Figure S1. Literature research process - first review (effect of keratinized mucosa).

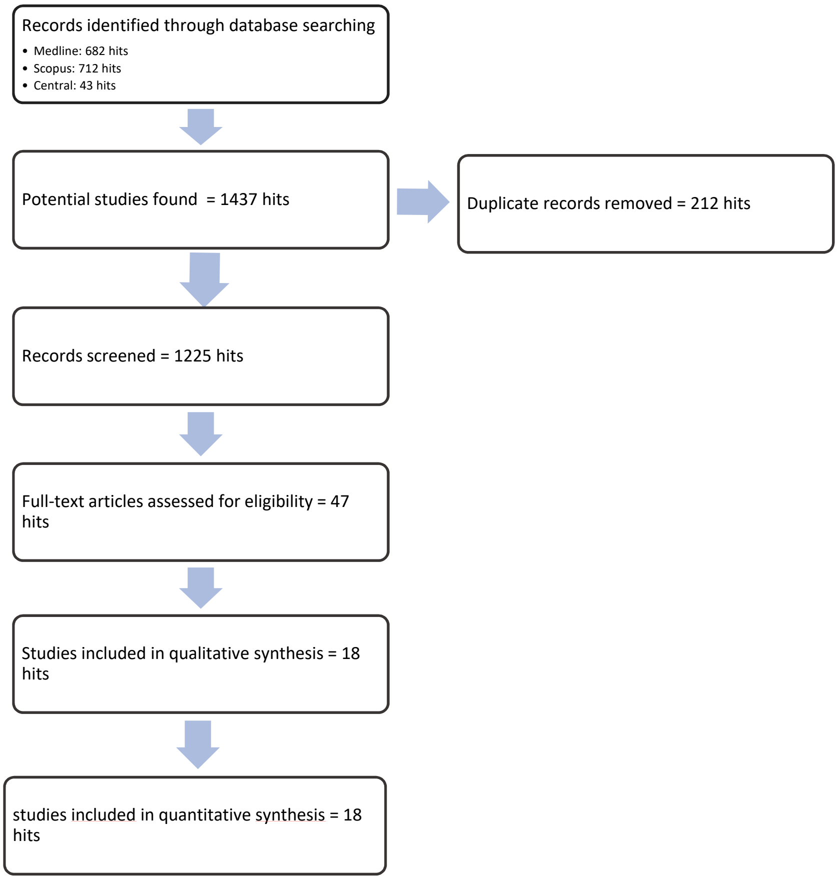

Figure S2. Literature research process - second review (effect of matrices vs autogenous grafts).

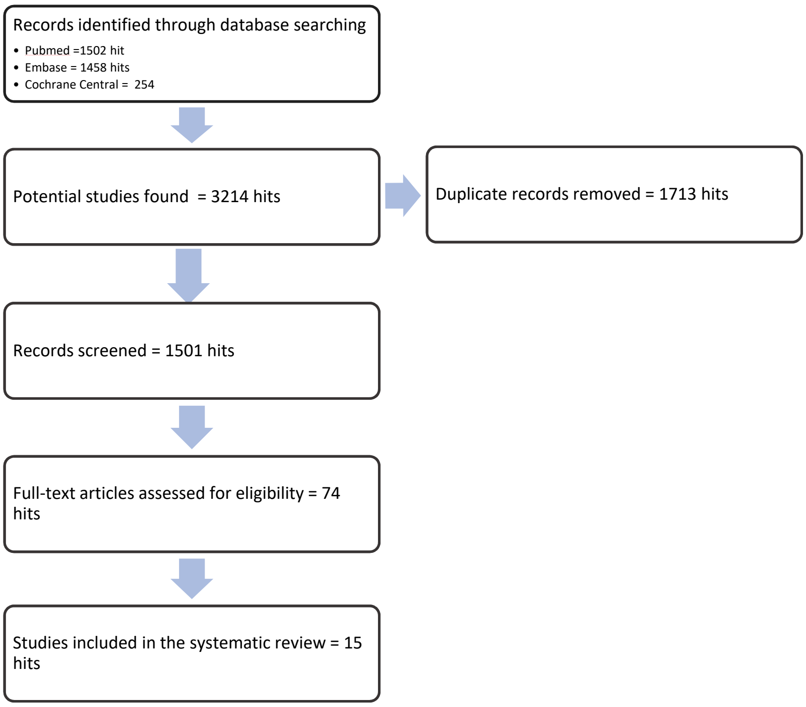

Figure S3. Literature research process - third review (effect of the abutment materials).

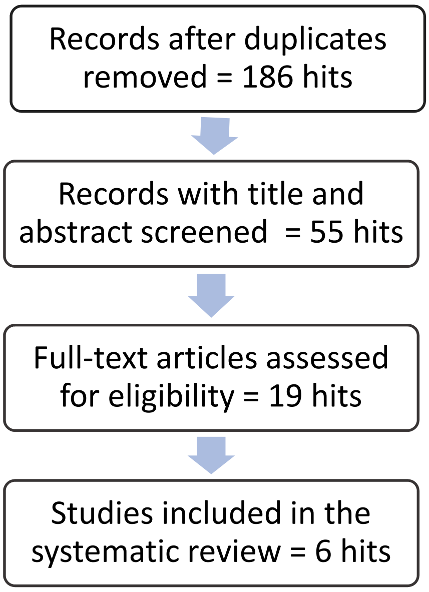

Figure S4. Literature research process - fourth review (effect of soft tissue thickness on peri-implant bone loss).

Table S2. limits of each review.

Limits of review #1 (effect of KM)

- Cross-sectional and prospective control studies were included
- The analysis was performed at the implant level
- Potential confounding factors were presented in some studies: the Authors of the review were not able to analyze their influence on the effect of the presence or absence of KM

Limits of review #2 (effect of matrices vs autogenous grafts)

- Limited number of included studies
- High heterogeneity among studies (number of patients and sites treated; the surgical techniques and different timing used for the soft tissue augmentation; the outcomes investigated; the methodology used to measure the values; and the variability in the follow-ups)
- The presence of risk factors contributing to potential inaccuracies in the outcomes

Limits of review #3 (effect of the abutment materials)

- Heterogeneity among the included studies, particularly in the MBL and PI analyses, regarding the study's design and clinical procedures
- Some of the included studies were primarily designed to assess aesthetics, not bone loss.
- Follow-up from 6 months to 7 years in the different studies
- Lack of clinical subgroups (e.g., according to the initial soft tissue quality and dimension),

Limits of review #4 (effect of soft tissue thickness on peri-implant bone loss)

- Half of the included studies were performed by the same scientific group
- Studies included different types of dental implants and prosthetic abutments with different geometry and shape
- The different positioning of the im- plants (crestal or sub-crestal)
- The use of a periodontal probe to assess soft tissue thickness
